# Integrated Palliative Care and Oncologic Care in Non-Small-Cell Lung Cancer

**DOI:** 10.1007/s11864-016-0397-1

**Published:** 2016-03-31

**Authors:** Divya Chandrasekar, Erika Tribett, Kavitha Ramchandran

**Affiliations:** Hospice and Palliative Medicine, Stanford University School of Medicine, 2502 Galahad Court, San Jose, CA 95122 USA; General Medical Disciplines, Stanford University School of Medicine, Medical School Office Building, 1265 Welch Road, MC 5475, Stanford, CA 94305 USA; Outpatient Palliative Medicine, Stanford Cancer Institute, Medical School Office Building, 1265 Welch Road MC 5475, Stanford, CA 94305 USA

**Keywords:** Non-small-cell lung cancer, Palliative, Quality of life, Lifestyle, Psychosocial, Symptom, Spiritual, Caregiver, Geriatric, Survivorship, Value, Multidisciplinary

## Abstract

Palliative care integrated into standard medical oncologic care will transform the way we approach and practice oncologic care. Integration of appropriate components of palliative care into oncologic treatment using a pathway-based approach will be described in this review. Care pathways build on disease status (early, locally advanced, advanced) as well as patient and family needs. This allows for an individualized approach to care and is the best means for proactive screening, assessment, and intervention, to ensure that all palliative care needs are met throughout the continuum of care. Components of palliative care that will be discussed include assessment of physical symptoms, psychosocial distress, and spiritual distress. Specific components of these should be integrated based on disease trajectory, as well as clinical assessment. Palliative care should also include family and caregiver education, training, and support, from diagnosis through survivorship and end of life. Effective integration of palliative care interventions have the potential to impact quality of life and longevity for patients, as well as improve caregiver outcomes.

## Introduction

Lung cancer is a devastating disease and is the leading cause of cancer-related mortality in the USA [1]. The last few decades has shown an accelerated rise in technologies and therapies in non-small-cell lung cancer (NSCLC) treatment. Current oncology care focuses on treatment choices that include radiation therapy, interventional procedures including surgery, and systemic therapy [2]. Despite these advances, patients with NSCLC face many challenges that are not addressed by our current model of care. NSCLC patients experience high symptom burden, rapidly declining functional status, social decline, psychological symptoms including emotional and spiritual angst, and often confront a poor prognosis.

Palliative care focuses on relief of symptoms across the physical and psychosocial domains and promotes shared decision-making. Palliative care services should also include relief and support for family members and caregivers. Although traditionally implemented late in a patients’ disease course, palliative care is gaining momentum and has the potential not only to affect a patients’ quality of life but also to significantly alter disease trajectory, and may even prolong survival [3, 4••, 5]. Services must be rendered to patients across the spectrum of disease and fine-tuned according to a patient’s individual needs. Importantly, as the world moves toward delivering more fiscally responsible, high value care, the integration of palliative care into oncologic care has the potential for improving quality of care as well [6–9].

The purpose of this review is to take a comprehensive look at the relevant published studies since 2000 to highlight some of the main advances and potential impact of palliative care integration into the treatment of non-small-cell lung cancer (NSCLC). This data will be used to provide recommendations on palliative care interventions to be included as part of care pathways for patients with NSCLC.

## Palliative therapies

### Physical symptoms

Physical symptom burden in NSCLC patients is high and negatively impacts quality of life [10, 11•, 12]. Pain, dyspnea, cough, anorexia/cachexia, and fatigue are the five most common and distressing symptoms [13]. Patients with early-stage lung cancer may experience minimal symptoms; however, as the disease becomes more advanced, symptoms are more prevalent. An integrated palliative approach to care would include validated instruments to measure symptom severity routinely. Subsequent referral to specialist care when symptoms become more severe or complex is recommended.

Potential palliative therapeutic approaches to assessing and managing these common symptoms are outlined below.

#### Exercise

Fatigue is a common symptom experienced by the NSCLC population that has an impact on the ability to exercise and maintain functional status. Individuals with NSCLC engaged in less physical activity to similar aged healthy individuals. They also had higher levels of depression, spent less time outdoors, and had lower motivation to engage in exercise [14]. Patients who have undergone lung resection therapy show deterioration in walking distance [15]. Patients with inoperable lung cancer undergoing chemotherapy tend to be more debilitated [16].

Based off growing data, promotion of physical activity is justified, feasible, and well tolerated during and after cancer therapy. Interventions, both pre- and post-operatively, have shown positive benefits including in cancer-related fatigue severity, emotional well-being, functional status, and overall quality of life. Patients who were referred earlier in the course of their treatments were more likely to adhere to and tolerate physical activity [17•, 18, 19]. In the post-surgical setting, several studies have demonstrated the positive effects of exercise programs on endurance and other quality of life parameters [20–23]. Therefore, early referral to physical therapy for tailored physical activity recommendations should be incorporated into NSCLC patient care.

#### Nutrition

Patients with lung cancer often experience anorexia and cachexia [24–26]. Together, these constitute the cancer anorexia–cachexia syndrome (CACS), a state of muscle catabolism and weight loss experienced as appetite loss [27].

Weight loss and malnutrition have been associated as independent risk factors for post-surgical complications such as intensive care support and post-operative death [28–30]. Data suggests that weight loss during concurrent chemoradiation therapy for NSCLC starts early and prior to onset of symptoms [31]. These symptoms may also have a prognostic impact on patients receiving chemotherapy in advanced disease [32].

Additionally, in advanced disease, the impact of these symptoms can be profound and can impact ability to deliver appropriate disease-modifying therapies [33, 34]. It has also been noted that people with advanced cancer are often found to have fatty acid deficiency [35], which in various studies has been shown to affect nutritional status, functional status, physical activity, and quality of life in lung cancer patients during multimodality treatment [36, 37].

Research suggests that interventions such as assessment of nutritional imbalances and supplementation with appropriate nutrients should be done early and reevaluated throughout illness trajectory [38, 39]. The use of validated assessment instruments such as the FAACT scale in patients with CACS should be utilized to standardize management and follow-up effects of nutritional interventions [40–42].

#### Pain management

Approximately 75 % of all cancer patients live with chronic pain. Pain is the most common symptom for patients with NSCLC, and they experience acute and chronic pain largely in the chest and lumbar regions [43–45]. Chest pain is present in an estimated 20 % of patients with lung cancer, and this pain increases in severity as the disease progresses [46]. Pain is multifactorial, and thus, often requires a multidisciplinary approach to successful management [47, 48].

The World Health Organization’s (WHO) Analgesic Ladder for Cancer Pain Relief is a cornerstone guide for pain management for patients with cancer, regardless of diagnosis. The ladder describes pain management in a series of steps as follows: (1) the use of non-steroidal anti-inflammatory drugs (NSAIDs); (2) weak opioid analgesics; and (3) strong opioid analgesics such as morphine, oxycodone, or fentanyl [49]. Breakthrough pain, or a temporary intensification of pain during opioid therapy, may occur and is usually alleviated with immediate release opioids such as fentanyl [47]. Adjuvant analgesics such as anti-depressants and anti-convulsants can supplement opioid use at any stage [50].

While opioids are the mainstay of pain management, special considerations must be taken to mitigate side effects. Titration and monitoring is critical to account for tolerance and metabolic concerns, such as renal insufficiency. Additionally, meta-analyses have indicated that opioid use has resulted in constipation in an average of 41 % of patients. Thus, laxatives are recommended alongside opioid use to prevent opioid-induced constipation [51].

More complex cases of refractory pain during the use of analgesics have shown to benefit from interventional strategies. Radiofrequency ablation is one way to treat pain due to metastases. In one 2015 study of 12 patients with rib metastases, pain reported via a visual analogue scale decreased from 7.9 to 3.4 with no symptomatic complications [52]. For patients experiencing bone metastases, radiation therapy is indicated. While questions remain as to the optimal dose and schedule, approximately 40 % of lung cancer patients receive radiotherapy, and a majority of these are with palliative intent [53, 54]. Additionally, complementary therapies such as mind-body modalities, massage, and acupuncture have been accessed by up to 60 % of cancer patients and have shown a significant benefit [55].

#### Respiratory support

NSCLC patients often experience a respiratory symptom cluster characterized by dyspnea and cough, manifesting especially in advanced disease. The prevalence of dyspnea alone is estimated between 55 and 87 % throughout all stages of lung cancer [56–59]. Dyspnea correlates with coping capacity, performance status, and other symptoms such as anxiety, depression, fatigue, and cough [60]. Further, dyspnea has been shown to account for over 25 % of urgent care visits in a study of 114 patients with NSCLC, second only to pain [61].

Management of dyspnea relies on the immediate treatment of breathlessness with medication and oxygen/air therapy, as well as pulmonary rehabilitation including exercises, breathing retraining, and smoking cessation [58]. Studies have indicated that a combination of pharmaceutical treatment of underlying causes and longer-term therapies for symptom suppression are the most effective [62]. These strategies have been shown to reduce breathlessness, fatigue, and accompanying anxiety while increasing endurance, functional status, and quality of life [58].

Oxygen therapy is a standard of care for patients requiring respiratory support [63]. Portable oxygen or high-flow oxygen may be indicated based on a patient’s oxygen saturation levels. Patients who are not hypoxemic may receive similar relief of dyspnea using high-flow room air as high-flow oxygen [64].

In patients with refractory dyspnea who no longer experience benefits from portable or high-flow oxygen, opioids are indicated [65]. A review of the use of opioids in the management of dyspnea found that oral opioids, specifically systemic opioids such as once-daily morphine, could increase comfort and decrease the sensation of breathlessness [66, 67••].

Non-pharmacological therapies that have resulted in symptom management are largely based in exercises for endurance and breathing efficiency, management of psychological distress, and lifestyle modifications such as smoking cessation [67••]. Evidence suggests that exercises such as pursed lip and diaphragmatic breathing are effective in alleviating dyspnea [67••]. Activities to reduce distress and improve self-efficacy include breathing exercises, relaxation techniques, and psychological support provide an additive and lasting therapeutic effect while promoting self-efficacy in managing dyspnea [67••, 68].

### Psychosocial distress

Palliative care should not only address disease-specific physical complaints and symptoms but should also include assessment of psychological, social, spiritual, and financial well-being [69, 70]. Psychosocial status appears to mirror physical decline, particularly surrounding key transition points in a patient’s disease course; namely, at time of diagnosis, discharge after treatment, as disease progresses, and at end of life. These may be the time periods to focus palliative care interventions [71].

For example, it is known that depression and anxiety are common and persistent symptoms experienced by patients diagnosed with NSCLC ranging in prevalence between 25 and 50 % and persistent in approximately 50 % of patients, especially among those with more severe symptoms or functional limitations [72–74]. There have been several studies linking the presence of depression as a predictor of worse survival in cancer, including in newly diagnosed metastatic NSCLC [75–77]. Another study suggests that the presence of anxiety, depression, and worse psychological quality of life during early stage of treatment was associated more with receipt of chemotherapy at the end of life [78]. A patients’ specific coping style may be impacted by their mental health and likely has repercussions on medical decision-making and disease trajectory [79, 80].

Comprehensive validated assessment instruments to measure psychological distress such as the distress thermometer should be used. In addition to screening for depression and anxiety, other areas of concern include family relationships, emotional functioning, lack of information regarding diagnosis/treatment, and practical problems such as financial issues or child care [81]. Referrals to appropriate specialists, which include social workers, specialized nurses, care coordinators or case managers, financial specialists, mental health professionals or spiritual counselors should then be made based on initial assessment.

#### Smoking cessation

Although decreasing, approximately 16.8 % of the adult US population still smokes cigarettes [82]. Smoking is a known risk factor for NSCLC with current smoking status conferring a worse overall survival and disease-free survival [83]. Smoking is also a known factor associated with increased risk of developing mutations such as KRAS and p53 known to be associated with NSCLC [84, 85]. Chronic nicotine exposure impacted response to therapy in EGFR-mutated NSCLC patents receiving targeted therapy, thus impacting disease trajectory [86]. An association between smoking and the development of NSCLC-COPD has been studied [87].

Furthermore, studies have shown that second primary lung cancers were rare in non-smokers but were associated with increased risk of occurring with increased tobacco exposure. In contrast, non-smokers, former smokers, and recent quitters showed a significant better prognosis [88]. Data also exists that links longer time since cessation of smoking to improve survival outcomes for NSCLC patients with early disease [89]. Patients who quit smoking after the diagnosis of NSCLC maintain better performance status [90]. Given such overwhelming evidence, it is clear that smoking cessation should be prioritized in the care of all patients, and especially in the NSCLC population.

Smoking cessation is more effective with a combined non-pharmacologic and pharmacologic approach. The National Cancer Institute recommends a five-step behavior change process which includes asking the patient about their smoking status, assessing their willingness to make a quite attempt, advising them to stop, assisting them with cessation efforts, and arranging follow-up. Individual and/or group therapy is often appropriate in addition to nicotine replacement therapy, which is the mainstay of pharmacologic treatment. Other medications such as bupropion and varenicline are available, as well as nortryptiline and clonidine, which are available off label for this purpose. Routine follow-up is recommended as the more extensive the support, the higher the likelihood of successful cessation [91–93].

### Spiritual health

Palliative care interventions should include content that targets the spiritual needs of patients, their family members, and caregivers. Spirituality is multidimensional, encompassing constructs such as faith and meaning, which may or may not be connected to religious affiliations [94]. Religiousness and spirituality improved depressive symptoms and may ease end of life distress. Women in particular may benefit from spiritual support as they reported more intense problems with emotional functioning and showed benefit from supportive spiritual care [95, 96]. Attempts should be made for providers to use validated assessment instruments such as the FICA to quantitatively measure and track spiritual well-being through treatment course and afterward [97]. Dependent on results of a needs assessment, engaging spiritual care specialists should be considered.

## Considering the continuum

### Early-stage disease pathway and survivorship

Patients with early-stage NSCLC have disease limited either to the lung (0, IA, IB) or local spread to lymph nodes located on the same side of the chest to where the cancer started (IIA). When possible, early-stage disease is treated with surgery to resect the tumor with 5-year survival approximated at approximately 50 % [98].

Given that surgery is the mainstay of therapy for this stage of disease, perioperative risk stratification and risk modification are important. Assessment of physical symptoms, psychosocial distress, spiritual distress, and caregiver distress should be a common practice for all. However, focus on nutritional and exercise therapy interventions, along with support for smoking cessation, are the mainstay of palliative therapy for this stage. Clinicians and patients should participate in shared decision-making with the initiation of advance care planning efforts.

A survivorship plan should also be developed for patients and families. Survivors face significant challenges post-treatment. Fatigue and respiratory symptoms are the most prevalent physical symptoms among NSCLC survivors and are associated with functional impairment. A comprehensive approach to treatment of these symptoms also includes management of anxiety, depression, and pulmonary disorders [99]. Structured exercise programs can improve physical functioning, mood, respiratory distress, and sleep efficiency [100–102]. Referral to physical activity and pulmonary rehabilitation programs may promote higher quality of life outcomes [103].

Other concerns for lung cancer patients post-treatment include psychological distress, depression, financial issues, and fear of recurrence of NSCLC or secondary malignancies [104••]. Psychosocial support, including mental health support, spiritual support, caregiver support, and financial advice, should be offered alongside continued medical management of symptom sequelae.

Adherence to recommendations on surveillance for recurrence and second primary lung cancers should be followed. The majority of second primary lung cancers and recurrences were detected by post-therapeutic surveillance computed tomography (CT) scans. The highest risk of recurrence persisted in the first 2–4 years following resection with curative intent [105–107]. The American Association for Thoracic Surgery recommends that long-term lung cancer survivors have low-dose CT to detect second primary lung cancer until the age of 79 years [108].

### Locally advanced disease pathway

For patients with locally advanced disease, treatment can be complex and includes a combination of radiation therapy, systemic therapy, and surgical interventions, often in sequence or combination [109]. As such, in addition to the standard clinical assessments and palliative interventions above, there is a greater need to coordinate a larger multidisciplinary team often involving medical oncologists, radiation specialists, and surgeons. Discussion of cases in multidisciplinary team meeting forums has shown evidence for better treatment tolerability and outcomes [110]. The evidence from several studies suggest that earlier integration of specialist palliative care team in this collaborative team environment is feasible and has a beneficial impact on symptoms and other quality of life measures [111, 112, 113••].

### Advanced disease pathway

The goal of treatment in advanced disease is prolongation of quality of life in the context of patient goals and values. Family and caregiver distress is often more acute, and the need for training, education, and support through treatment, end of life, and bereavement is critical. The more common therapeutic strategies in this setting include radiation for the purposes of palliation of symptoms and systemic therapy.

Radiation therapy has shown associated improvements in several physical symptoms including pain, dyspnea, cough, and more moderately in fatigue and appetite loss [114–116]. Overall improvements in physical, cognitive, social, and emotional functioning were noted, along with beneficial effects on overall global quality of life [117, 118].

The mainstay of treatment is systemic and includes chemotherapy, immunotherapy, and targeted agents, used to improve quality of life. There is a strong relationship between response to chemotherapy and survival when compared to best supportive care [119, 120]. Additionally, palliative chemotherapy has been associated with improvements in several components of symptom relief and quality of life measures [121–123]. Having said this, continuing chemotherapy for advanced NSCLC until very near death is associated with a decreased likelihood of receiving hospice care but not prolonged survival [124]. Beyond three courses of chemotherapy conveys no survival or consistent quality of life benefits in advanced NSCLC patients [125]. Oncologists should strive to discontinue chemotherapy as death approaches and encourage patients to enroll in hospice for better end of life palliative care.

Considering this evidence, involvement of palliative care specialists is warranted, given the added mental, emotional, psychosocial, and medical complexity of care at this stage of disease. Importantly, involvement of palliative care specialists is beneficial to find the appropriate timing of transition to hospice care, which is often accomplished too late in the current model of care. In this one study, patients with metastatic NSCLC who received early palliative care measures still received similar number of chemotherapy regimens, but the timing of final chemotherapy administration and transition to hospice services were optimized [126].

## Special considerations

### Lung cancer in geriatric populations

The age at diagnosis for lung cancer is most often >70 years old, and oncologists are becoming increasingly aware of this expanding aged cancer population who face unique physical, psychological, and social challenges when receiving care. However, it is also becoming acutely apparent that similar to younger patients, carefully selected elderly patients can benefit from therapy at all stages of disease, and should not be excluded or under treated on the basis of age alone [127–131].

There is no significant difference in response or toxicity between younger and elderly patients receiving palliative radiation therapy [132]. Furthermore, preliminary studies show that higher doses of radiation are safe, effective, and may affect survival in aged NSCLC patients [133]. The previous fear that chemotherapy would be particularly toxic and harmful to elderly patients has also been quelled [134]. As with any age, consideration of comorbid conditions and risk factors should be weighed but elderly patients should not be excluded from this option [135]. Even patients with poor prognostic factors still exhibit positive response and symptom relief with palliative chemotherapy [136, 137]. Palliative surgical treatments too are being considered more readily in the elderly given similar benefits and quality of life expectations [138, 139]. As with other age groups, geriatric populations want to be involved in the decision-making process.

### Family and caregiver support

Caregivers experience high levels of caregiver burden and reported deterioration in psychological well-being and overall quality of life [140, 141]. Distress and anxiety are understudied but accepted as widely prevalent among patients and their family caregivers. Several common areas of anxiety and distress exist, with the most prevalent themes emerging as uncertainty, loss or impending loss, changing roles, conflict outside, finances, physical symptoms, fears of decline and dying, and life after the patients’ passing [142].

A large area of distress stems from caregiver management of patient symptoms. There may be a discrepancy in how lung cancer patients and their caregivers assess and address both psychological and physical symptom occurrence and distress [143, 144]. Patient and caregiver self-efficacy and congruence of self-efficacy for managing pain, symptoms, and function affect several factors of distress and overall quality of life [145–147].

When given supportive services, both patients and caregivers have shown improvements in various parameters including pain, depression, self-efficacy, anxiety, and overall quality of life [148]. Thus, palliative care interventions aimed at caregiver and patient education, training, and support should be implemented early and consistently. Supportive efforts should be continued through treatment into the survivorship phase, and/or into the bereavement phase.

### Advance care planning and transitions in care

Advance care planning (ACP) is a central component of palliative care. It includes both the medical aspect of care involving discussions with patients and families regarding goals of care, and the legal aspect of care involving the completion of either an advance directive form or a POLST. Presently, there are poor completion rates for ACP [149]. Despite overwhelming evidence that involvement of palliative services and hospice improve quality of care [150–152], poor utilization remains [153]. The goal would be to engage specialist palliative care teams earlier in a patient’s illness trajectory in order to maximally utilize resources while prioritizing shared decision-making and patient values.

## Limitations

**Fig. 1:**
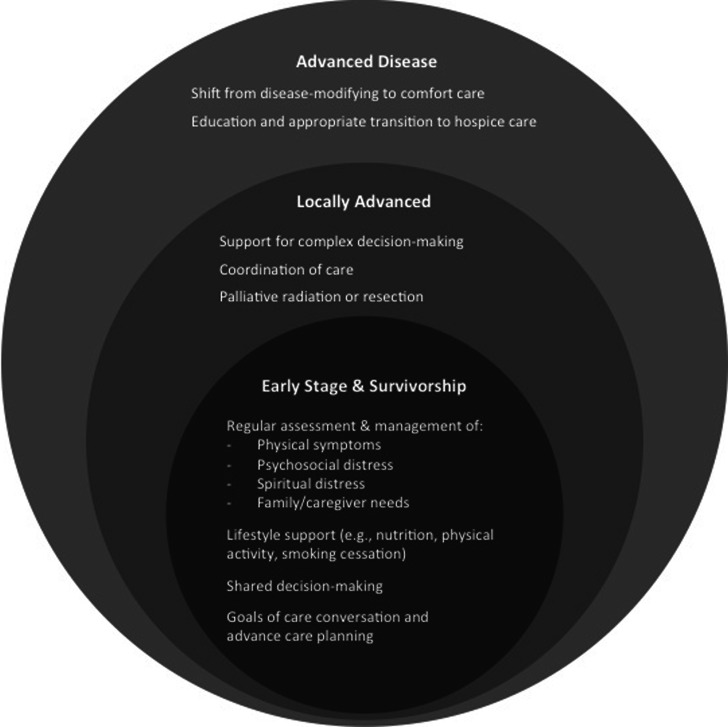
Summary of the palliative needs throughout the continuum of care for patients with NSCLC.

Research using quality of life parameters as outcome measures is becoming more accepted and widespread. However, most studies evaluating palliative measures in NSCLC have thus far been small and with mixed results. Research needs to be reproduced and replicated in order to validate a standardized approach to comprehensive cancer care for patients with NSCLC that integrates palliative care as a mechanism for ensuring optimal quality of life at any stage of disease experience (Fig. 1).

## Conclusions

Palliative care is a specialized field that aims to provide effective symptom management and to maximize quality of life for patients and their caregivers through the entirety of illness trajectory from diagnosis to survivorship and end of life. Utilizing the care pathways as described in this article allow for personalized screening, assessment, and consistent implementation of palliative care interventions for patients and their families.

Initial treatment plan for all patients, regardless of stage, should encompass early assessment of clinical domains including physical symptoms, psychosocial distress, spiritual distress, and caregiver distress. Subsequent implementation of interventions and specialist referrals should be made based off needs of patients and caregivers.

In early-stage disease, the focus is primarily on surgical risk assessment and modification with particular attention paid to nutritional interventions and implementation of exercise training programs. It is essential that in post-therapy, there is utilization of a survivorship team to assist with treatment sequelae. In locally advanced disease, specific attention should be paid to coordination of care in addition to managing the physical ramifications of complex treatments such as concurrent chemotherapy and radiation and/or complex surgery. In the advanced stage of disease, relief of symptoms and optimization of care that is in alignment with patient and family goals is paramount. Additionally, understanding when a systemic treatment is appropriate for palliation and longevity versus when it may be contributing to hastening end of life is important. At this stage, it is recommended to add a specialist palliative care team to assist with ACP and transition to hospice. Palliative care is an expansive and innovative field that has the potential to revolutionize patient care.
